# Huangshui Polysaccharide Exerts Intestinal Barrier Protective Effects through the TLR4/MyD88/NF-*κ*B and MAPK Signaling Pathways in Caco-2 Cells

**DOI:** 10.3390/foods12030450

**Published:** 2023-01-18

**Authors:** Jiaying Huo, Wenhao Pei, Guoying Liu, Weizheng Sun, Jihong Wu, Mingquan Huang, Wei Lu, Jinyuan Sun, Baoguo Sun

**Affiliations:** 1 Key Laboratory of Brewing Molecular Engineering of China Light Industry, Beijing Technology and Business University, Beijing 100048, China; 2 Beijing Laboratory of Food Quality and Safety, Beijing Technology and Business University, Beijing 100048, China; 3 School of Food Science and Engineering, South China University of Technology, Guangzhou 510640, China; 4 Anhui Gujing Distillery Co. Ltd., Bozhou 236820, China

**Keywords:** Huangshui, *α*-D-glucan, intestinal barrier protection, TLR4/MyD88/NF-*κ*B, p38 MAPK, RNA-seq

## Abstract

Several reports have demonstrated that natural polysaccharides exert protective effects on intestinal barrier function. In our previous study, we isolated a polysaccharide named HSP-W from Huangshui (HS). In the present study, the protective role of HSP-W in LPS-induced intestinal barrier dysfunction was determined by several molecular biological techniques. The results showed that HSP-W treatment alleviated the deduced TEER and increased the permeability of intestinal epithelial cells induced by LPS through inhibiting the release of inflammatory cytokines and enhancing the expression of tight junction (TJ) proteins. The underlying molecular mechanisms were elucidated by RNA-seq technique, which indicated that the differentially expressed genes (DEGs) between the LPS-treated and LPS+HSP-W-treated groups were enriched in the “MAPK” signaling pathway. Notably, the overlapping DEGs reversed by HSP-W intervention highlighted the pathways of the “Toll-like receptor” and “NF-*κ*B” signaling pathways. The suppression of p38 and NF-*κ*B were mediated by the inhibition of MyD88. Furthermore, HSP-W treatment prevented the translocation of NF-*κ*B to nucleus, thus inhibiting the release of TNF-*α*, IL-6, and IL-1*β*. Overall, HSP-W has beneficial effects on LPS-induced inflammation; it protects the intestinal barrier from injury in Caco-2 cells through inhibiting the TLR4/MyD88/NF-*κ*B and p38 MAPK signaling pathways.

## 1. Introduction

In addition to being the main place for nutrient digestion and absorption, the gut also maintains homeostasis as a barrier [1], including physical barrier, chemical barrier, immune barrier, and biological barrier [2]. Among them, the physical barrier, which consists chiefly of tight junctions (TJs) and intestinal epithelial cells, is the first defensive line of the intestinal tract, which can prevent the passage of endotoxins, harmful bacteria, and foreign antigens while allowing the absorption of nutrients, water, and electrolytes [3,4]. However, it has been reported that intestinal barrier damage is manifested by mucosal inflammation and increased intestinal permeability [5,6], which is an important cause of the occurrence and development of inflammatory bowel diseases (IBD), including ulcerative colitis (UC) and Crohn’s disease (CD) [7]. Therefore, improving intestinal barrier function is of great significance for the treatment of IBD [8]. At present, the main treatment for IBD is to take aminosalicylate, corticosteroids, immunomodulators, and other drugs, but long-term use of these drugs will cause serious side effects, including increased susceptibility to malignant tumors and infections [9]. There have been many studies showing that natural active substances from foods or plants, such as flavonoids [10], polyphenols [11], polysaccharides [12], and bioactive peptides [13], can play an anti-inflammatory role in vitro using Caco-2 cells. At present, the Caco-2 monolayer cell model is widely used to study the integrity and function of the intestinal epithelial barrier [14]. Lipopolysaccharide (LPS), the most used differentiation Caco-2 monolayer stimulator, can trigger the inflammatory response of Caco-2 cells in vitro, simulating intestinal epithelial barrier dysfunction [15]. The integrity of TJ in the epithelial monolayer can be represented by quantifiable indicators, transepithelial electrical resistance (TEER), and paracellular flux of fluorescein isothiocyanate dextran 4 (FD-4) or phenol red, to determine the extent of intestinal barrier damage [16].

Previously, a polysaccharide named HSP-W was isolated and purified from Huangshui (HS), which is an important by-product of the brewing stage of Baijiu production [17]. It has the characteristics of large output and high pollution, and has been used to cultivate low value-added industries, such as artificial pit mud, thanks to its high content of various organic compounds and microorganisms [18]. According to our previous research, HS is rich in polysaccharides, which have health effects, such as immunity and antioxidation [19,20]. Huangshui polysaccharides were reported to stimulate the production of ROS, NO, TNF-*α*, and IL-6, up-regulate the mRNA and protein expression levels of TNF-*α* and IL-6, and enhance the pinocytic and phagocytic capacities of THP-1 cells [20]. In addition, they show antioxidant activity in vitro by scavenging DPPH radical and ABTS radical [19]. HSP-W was a homogeneous glucan with a molecular weight of 166.00 kDa and composed of a 1,4 linked *α*-D-Glc*p* backbone with the substitution at O-6 with 1,6-linked *α*-D-Glc*p* residue and non-reducing terminal of *β*-Glc-1 [17]. It is reported that glucans from marine fungus *Phoma herbarum* YS4108164 [21] and yeast [22] can alleviate intestinal barrier damage and improve mouse colitis through restraining the release of inflammatory cytokines and up-regulating the TJ protein expressions related to intestinal integrity. In addition, a glucan from spore of Ganoderma lucidum inhibited apoptosis and up-regulated the expression of TJs (ZO-1, E-cadherin, *β*-catenin, and Occludin) to protect the intestinal barrier function in vivo and in vitro [23]. However, the intestinal barrier function of HSP-W has not been studied, especially the underlying mechanism remains unclear.

Thus, our research aimed to find out the intestinal barrier protective effects of HSP-W on LPS-induced Caco-2 cells and explore the underlying molecular mechanisms, with hope to provide valuable reference for the high-value utilization of HS.

## 2. Materials and Methods

### 2.1. Samples and Chemical Reagents

The HS polysaccharide (HSP-W) used in the present study was prepared from Gujinggong HS (Anhui Gujing Distillery Co., Ltd., Bozhou, China) according to our previous report [17], the structure (possible repeated unit) of HSP-W was displayed in Supplementary Figure S1. The other chemicals used in the present study are listed in the supporting information file.

### 2.2. Cell Culture and Cell Viability Analysis

After purchase from the Cell Bank of Wuhan University (Wuhan, China), the human colon cancer cell line Caco-2 was cultured in DMEM (containing 4500 mg/L glucose) with 10% fetal bovine serum in a humidified incubator containing 5% CO_2_ at 37 °C.

The effects of HSP-W and LPS on Caco-2 cell viability were determined by CCK-8 assay [24]. Briefly, Caco-2 cells were treated with various concentrations of LPS (1.0~50.0 μg/mL) or HSP-W (10.0~2000.0 mg/L), pre-incubated for 24 h in a humidified incubator containing 5% CO_2_ at 37 °C, and then 10.0 μL CCK-8 solution was added to each well, followed by continuous culturing for 4 h in 96-well plates in the incubator. Finally, the OD values (450 nm) were recorded with a microplate reader (SpectraMax M2e, Molecular Devices Corporation, San Jose, CA, USA), and the cell viabilities were calculated on the basis of the instructions.

### 2.3. Barrier Integrity and Permeability Assay

In the present study, we assessed the integrity and permeability of the intestinal barrier by measuring TEER values and FD-4 fluxes. Before the test, Caco-2 monolayer cells pre-cultured in Transwell chambers with TEER value of 400 Ω cm2 (after 14–21 days) were exposed to 50 μg/mL of LPS for 1–8 h, at the same time with HSP-W or after 24 h of HSP-W treatment (100, 250, and 500 μg/mL, the range of concentration was selected based on most reports in the literature about the polysaccharides with intestinal barrier activity [8,25,26] and the results of cell viability experiments). The TEER of Caco-2 was monitored by a Millicell-ERS volt-ohmmeter (Millipore, MA, USA) and the value was expressed as a percentage relative to the initial TEER. For the permeability test, FD-4 was added to the apical chamber at a final concentration of 100 μg/mL. Then, Caco-2 cells were incubated at 37 °C for 1–8 h, and subsequently 100 μL of the solution in the basolateral chamber was collected. After dilution with 100 μL of 1 × HBSS, the fluorescence intensity of the diluted solution in λex 485 nm and λem 520 nm was measured using a microplate reader.

### 2.4. ELISA Kit Assay

Caco-2 cells (1 × 10^5^ cells/mL) were cultured in 24-well plates at 37 °C and 5% CO_2_ for 24 h before measuring the proinflammatory cytokine levels. Caco-2 cells were treated with high, medium, and low concentrations of HSP-W for 24 h, then with LPS (10 μg/mL) for 4 h. After that, the culture medium in all groups was harvested and centrifuged at 4000× *g* for 15 min to obtain the supernatant, in which the content of IL-1*β*, IL-6, and TNF-*α* were determined using marketed ELISA kits.

### 2.5. Western Blot Analysis

Caco-2 cells were treated with precooled RIPA protein extraction reagent, centrifuged at 4000× *g* and 4 °C for 15 min, and the supernatant was collected for detection. Protein concentration was determined by BCA protein detection kit (Beyotime, Shanghai, China). The quantified proteins were separated by SDS-PAGE and then transferred from the gel to polyvinylidene fluoride (PVDF) membranes (Millipore). Subsequently, the membranes were completely immersed in QuickBlock™ Blocking Buffer (Beyotime, shanghai, China) and shaken at room temperature for 50 min to block the blots. The primary antibodies (Abcam, Cambridge, UK) were then added and cultured overnight on a shaking bed at 4 °C. Additionally, the membranes were incubated at 25 °C for 30 min and washed with TBST for 5 times. After that, the membranes were incubated with the second antibody (Proteintech Group, Wuhan, China), diluted with 1% BSA-TBST at 25 °C for 60 min, and cleaned with TBST 6 times. Finally, ECL plus and chemiluminescence system (CLiNX Science, Shanghai, China) were used for visualization.

### 2.6. Evaluation of the Transcriptome

Caco-2 cells (1 × 10^5^ cells/mL) were inoculated in 100 mm plates and incubated for 24 h (37 °C, 5% CO_2_) and then pretreated with or without 250 mg/L of HSP-W for 24 h followed by 24 h 10 μg/mL of LPS treatment. These cells were divided into three groups: Con (the control group), LPS (the LPS group), and LPS_SG (the group pretreated with HSP-W and then LPS), and three samples of each group were carried out to ensure the reproducibility. In the present study, the total RNA extraction was conducted using TRIzol^®^ Reagent (Invitrogen, Waltham, MA, USA), and Qubit^®^ RNA Assay Kit (Thermo-Fisher, Waltham, MA, USA) was used to determine the RNA concentration. The RNA integrity verification was carried out by Agilent 2100 bioanalyzer (Agilent, Santa Clara, CA, USA) and the purity of RNA was verified using NanoPhotometer spectrophotometer (IMPLEN, Westlake Village, CA, USA).

Purified RNA (339.5–453.7 μg/mL, OD260/280: 1.93–2.01) was submitted to an Illumina Hiseq platform for sequencing and 150 bp paired-end reads were produced. Clean data with high quality reads were obtained after filtering. The obtained data were compared with the human genome reference GRCh38 from http://asia.ensembl.org/homo_sapiens/Info/Index (accessed on 1 December 2022). Data with an absolute value of log2|fold-change (FC)| > 0.58 and *p*-value < 0.05 (*t*-test) were identified as differentially expressed genes (DEGs), which required further gene ontology (GO) analysis and Kyoto Encyclopedia of Genes and Genomes (KEGG) enrichment analysis to obtain some related functional annotations and biological descriptions. Finally, the accuracy of the transcriptome was verified based on the results of validation on selected DEGs by RT-PCR method as described in Section 2.7 [27].

### 2.7. Real-Time Quantitative Polymerase Chain Reaction (RT-PCR)

After treatment as descripted in Section 2.5, the total RNA of Caco-2 cells was extracted according to the instructions of the RNA extraction kit, and the concentration and purity of RNA were detected by UV spectrophotometer (486.4–736.2 μg/mL, OD260/280: 2.05–2.08). After the synthesis of first-strand cDNA from 1 μg of RNA using the reverse transcription kit HiScript^®^ II Q select RT Supermix for qPCR (+gDNA wiper), qPCR detection was carried out by a CFX96 fluorescence quantitative PCR detection system (Bio-Rad) with a ChamQ Universal SYBR qPCR Master Mix Kit. The primer sequences (forward and reverse) required for the experiment were shown in supplementary Table S1. The mRNA expression levels were expressed by 2^−ΔΔCT^ method with GAPDH as the reference gene [20].

### 2.8. Nucleo-Cytoplzasmic Separation Assay

This part of experiment was carried out according to our previous study [27].

### 2.9. Statistical Analysis

All the data in this study were analyzed by SPSS for Windows (version 22.0, Chicago, IL, USA) and expressed as mean ± standard deviation (M ± SD). ANOVA and Duncan test were used to evaluate all the data. If *p* < 0.05, there was a significant difference between groups.

## 3. Results

### 3.1. Effects of HSP-W and LPS on the Caco-2 Cell Viabilities

**Figure 1:**
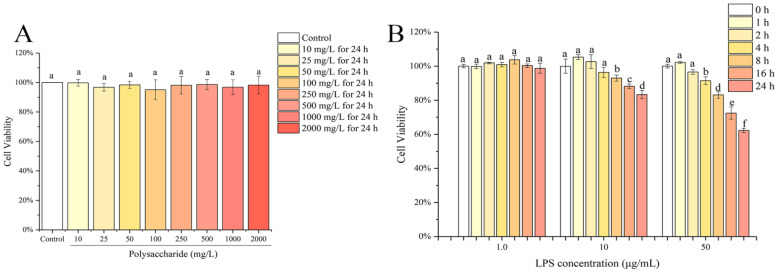
(**A**) Effects of HSP-W on Caco-2 cell viability, (**B**) effects of LPS on Caco-2 cell viability [28]. Different letters represent the significant difference at *p* < 0.05.

Before the activity test, the appropriate dosage of HSP-W and LPS that are non-toxic to cells needed to be determined by the CCK-8 assay. As shown in Figure 1A, compared with the control group, HSP-W in all tested concentration ranges had no significant effects (*p* > 0.05) on the cell viabilities, indicating that HSP-W had no toxicity to Caco-2 cells. The results in Figure 1B [28] showed that there was no obvious change in the viability of cells exposed to 1.0 μg/mL LPS for 24 h. However, when the LPS concentration increased to 10 μg/mL, the cell viability decreased significantly (*p* < 0.05) after 4 h of culture. Therefore, in the subsequent experiments, not only the LPS concentration but also the culture time should be considered to determine the appropriate LPS dose, which ensured cell viability above 80%.

### 3.2. Protective Effects of HSP-W on the Intestinal Barrier Function in Caco-2 Cells

**Figure 2:**
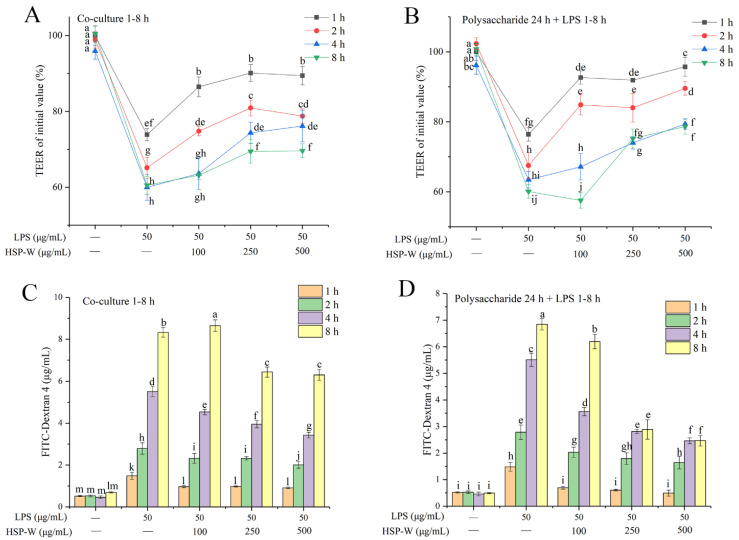
Effects of HSP-W on decreased TEER values and increased FD-4 fluxes caused by LPS: (**A**,**C**) co-culture with HSP-W for 1–8 h, or (**B**,**D**) pretreatment with HSP-W for 24 h. Data were shown as the mean ± SD from four independent experiments. Different letters represented the significant difference at *p* < 0.05.

In the present study, TEER values and FD-4 flux of Caco-2 cell monolayers treaded with or without HSP-W were determined to evaluate the integrity of the intestinal barrier. As illustrated in Figure 2A,B, LPS challenge reduced the TEER values from 100% to 73.87–59.98% after 1–8 h, respectively, while this decline was significantly reversed by co-culture with HSP-W for 8 h or pre-culture for 24 h. Our results displayed that pretreatment with HSP-W had better inhibitory effects on the decreased TEER by 0.00–68.86%, 32.21–65.77%, and 45.58–81.82%, at low, medium, and high concentrations, respectively. Furthermore, as displayed in Figure 2C,D, the FD-4 flux was elevated after LPS stimulation compared to the control group, but pre-culture or co-culture with different concentrations of HSP-W reduced the increase. Within 2 h, there was no significant difference (*p* > 0.05) in the effect of different concentrations of HSP-W on FD-4 flux, whereas after 2 h, especially when Caco-2 cells were exposed to LPS for 8 h, the high concentrations of HSP-W treatment were more effective than the low concentrations.

### 3.3. Inhibiting Effects of HSP-W on Inflammatory Cytokine Release Induced by LPS

**Figure 3:**
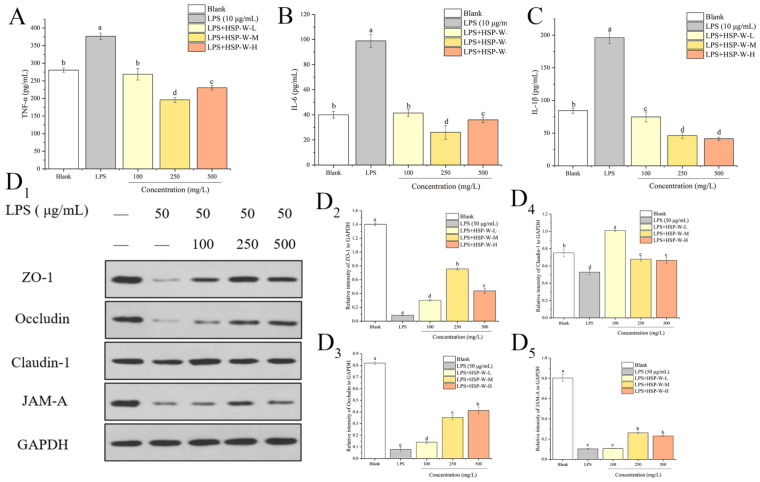
Effects of HSP-W on proinflammatory cytokines and TJ protein expressions in Caco-2 cells: (**A**) TNF-*α*, (**B**) IL-6, (**C**) IL-1*β*, and (**D**) TJ proteins. Data were shown as the mean ± SD from four independent experiments. Different letters represented the significant difference at *p* < 0.05.

As shown in Figure 3A–C, 10 μg/mL of LPS stimulation significantly induced the secretion of TNF-*α*, IL-6, and IL-1*β* by 34.33%, 147.15%, and 132.36%, respectively, compared to the control group (*p* < 0.05). However, treatment with different concentration of HSP-W (100, 250, and 500 mg/L) attenuated the release of inflammatory cytokines in LPS-challenged Caco-2 cells. Especially for the 250 mg/L of HSP-W pretreated cells, the secretion of TNF-*α*, IL-6, and IL-1*β* decreased by 47.99%, 73.65%, and 76.38%, respectively, compared with the LPS-treated cells, proving HSP-W reduced the inflammatory response induced by LPS.

### 3.4. Alleviation Effects of HSP-W on Tight Junction Proteins in Caco-2 Cells

Western blot was used to detect the expression of TJ proteins (Occludin, Claudin-1, ZO-1, and junction adhesion molecule-A (JAM-A)) to explore the protective mechanism of HSP-W against LPS-induced injury. Caco-2 cells were pre-cultured in Transwell chambers for 7-10 days until forming a cell monolayer. Thereafter, Caco-2 cell monolayers were treated with HSP-W (100, 250, 500 mg/L) for 24 h followed by exposing to 50 μg/mL of LPS in the apical chamber for 8 h. The results illustrated in Figure 3D indicated that compared with the control group, LPS stimulation caused obvious inhibition of Occludin, Claudin-1, ZO-1, and JAM-A by 9.59-, 0.42-, 15.27-, and 6.79-fold, respectively, whereas the decline was alleviated by pretreatment with HSP-W at different concentrations. Specifically, low, medium, and high levels of HSP-W up-regulated the expressions of Occludin, Claudin-1, ZO-1, and JAM-A from 81.29–434.67%, 13.57–74.62%, 252.19–777.04%, and 3.43–154.52%, respectively. We could obtain that the medium-level groups exerted better effect on protecting the LPS-damaged intestinal barrier function through increasing the expression of TJ proteins.

### 3.5. Visualization of DEGs

**Figure 4:**
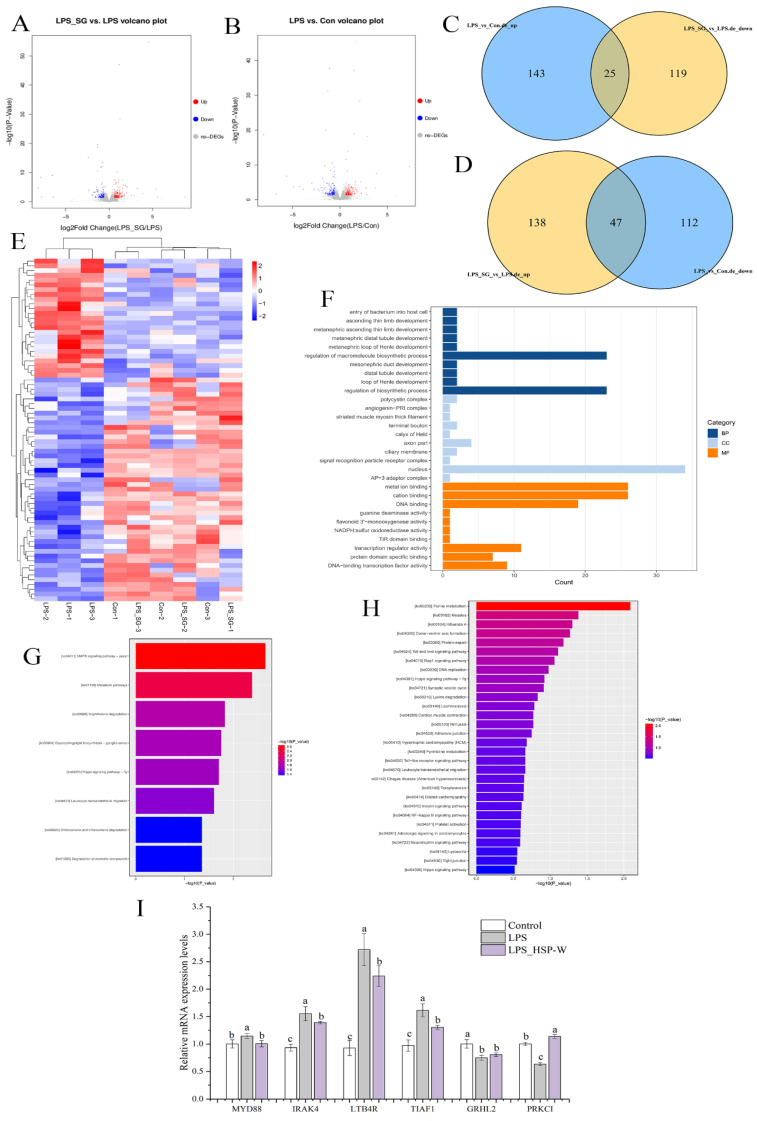
Transcriptomics analysis of effects of HSP-W on LPS-induced intestinal barrier injury. The DEGs are represented as a volcano figure. (**A**) LPS_SG vs. LPS, (**B**) LPS vs. Con, (**C**,**D**) Venn diagrams revealing DEGs regulated by HSP-W treatment, (**E**) heatmap revealing DEGs regulated by HSP-W treatment, (**F**) GO enrichment of the overlapping DEGs, KEGG enrichment of DEGs: (**G**) comparing the LPS_SG and LPS groups, (**H**) those reversed by HSP-W pre-incubation, (**I**) relative mRNA expression level of selected genes for verification. Different letters represented the significant difference at *p* < 0.05.

RNA sequencing (RNA-seq) was carried out to explore the mechanism underlying the protective effects of HSP-W on LPS-damaged intestinal barrier function in Caco-2 cells. LPS intervention caused the differential expression of 327 genes, of which 168 were up-regulated and 159 were down-regulated (Figure 4A–D). However, pretreatment with HSP-W changed the intervention. As shown in Figure 4A–D, a total of 329 DEGs were identified between the HSP-W and LPS groups (LPS_SG vs. LPS), of which 144 genes were down-regulated and 185 were up-regulated. Of note, 72 genes overlapped in the Venn diagram (Figure 4C,D) were reversed by HSP-W pretreatment, among them, 25 genes showed up-regulation after LPS stimulation were down-regulated after HSP-W treatment, while 47 genes displayed down-regulation after LPS stimulation were up-regulated by HSP-W intervention. Therefore, we speculated that these overlapped genes were therapeutic targets of HSP-W protection on LPS-challenged intestinal barrier injury. The heatmap in Figure 4E showed the overlapped DEGs that were reversed by HSP-W treatment.

### 3.6. GO and KEGG Analysis

Based on the above 72 overlapped DEGs, GO term enrichment analysis showed that many genes involved in the regulation of the macromolecule biosynthetic process, calcium-mediated signaling, transcription regulator activity, and DNA-binding transcription factor activity were differentially expressed, and these genes were mainly located in the nucleus, as shown in Figure 4F. Additionally, KEGG pathway enrichment analysis showed that the most important pathway involved in the protection of HSP-W on LPS-challenged intestinal barrier injury in Caco-2 cells was the MAPK signaling pathway (Figure 4G). Interestingly, 72 overlapped DEGs were enriched in the Toll-like receptor signaling pathway, NF-kappa B signaling pathway, adherens junction (AJ), and tight junction (Figure 4H), indicating that in addition to MAPK, the Toll-like receptor and NF-kappa B signaling pathways were of great significance in improving intestinal barrier function with HSP-W. Moreover, the genes associated with the AJs and TJs were regulated by HSP-W, proving that HSP-W might protect intestinal barrier function by regulating the expression of AJ or TJ proteins, which further confirmed the results in Section 3.4.

### 3.7. Verification of DEGs

The qPCR results shown in Figure 4I confirmed the changes in the expression of six selected genes, including four genes related to inflammation (MyD88, IRAK4, LTB4R, and TIAF1) and two TJ related genes (GRHL2 and PPKCI). HSP-W treatment obviously down-regulated the mRNA expression of MyD88, IRAK4, LTB4R, and TIAF1, those were up-regulated by LPS (*p* < 0.05). However, the TJ-related gene PPKCI was up-regulated with HSP-W pretreatment, and there was no significant difference in GRHL2 mRNA expression between the LPS group and the LPS_HSP-W group, which were consistent with the results of the transcriptome analysis, proving the accuracy and reliability of the transcriptome results.

### 3.8. Effects of HSP-W on TLR4/MyD88/NF-κB and p38 MAPK Signaling Pathways in Caco-2 Cells

**Figure 5:**
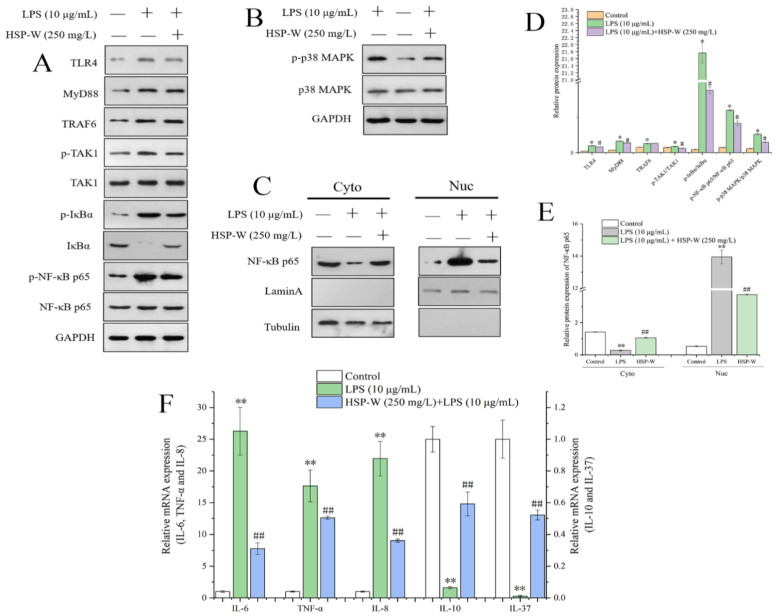
Effects of HSP-W on the TLR4/MyD88/NF-*κ*B and MAPK signaling pathways. (**A**) Western blot analyses of TLR4, MyD88, TRAF6, p-TAK1/TAK1, p-I*κ*B/I*κ*B, p-NF-*κ*B/NF-*κ*B, (**B**) p-P38/P38, (**C**) nucleo-cytoplasmic separation of NF-*κ*B, (**D**) densitometric quantification of the phosphorylated and total proteins, (**E**) densitometric quantification of NF-*κ*B in the cytoplasm and nucleus, (**F**) relative mRNA expression of proinflammatory and anti-inflammatory cytokines. The values represent the mean ± SEM (*n* = 3). * *p* < 0.05 and ** *p* < 0.01, compared with the control groups, # *p* < 0.05 and ## *p* < 0.01, compared with the LPS-treated cells.

The NF-*κ*B and MAPK signaling pathways were obtained in KEGG enrichment analysis. Hence, the key proteins of these two pathways were verified by Western blot. According to the results from Figure 5A,D, the protein expressions of TLR4 and MyD88 were notably up-regulated by LPS compared to the control group (*p* < 0.05), whereas HSP-W intervention down-regulated the increase and consequently the key phosphorylated adaptor protein p-TAK1 decreased significantly (*p* < 0.05). As a result, the ratios of p-I*κ*B/I*κ*B and p-NF-*κ*B/NF-*κ*B were both obviously decreased by 79.66% and 30.87%, respectively, compared to the LPS group (*p* < 0.05). In addition, the involvement of the NF-*κ*B pathway was explored by estimating the translocation to the nucleus of the transcription factor NF-*κ*B (Figure 5C,E). We could obtain that the expression of NF-*κ*B p65 in the cytoplasm decreased but, in the nucleus, increased under LPS treatment, leading to the promotion of IL-6, TNF-α, and IL-8 and the inhibition of IL-10 and IL-37 (Figure 5F). Compared to the LPS group, NF-*κ*B p65 expressions in cytoplasm and nucleus were significantly reversed by HSP-W and followed by the inhibition of proinflammatory factor and promotion of anti-inflammatory factor as illustrated in Figure 5F. Regarding the MAPK pathway, the increased level of p-p38/p38 was significantly reduced by HSP-W (Figure 5B). These results demonstrated that HSP-W could alleviate inflammation by suppressing the NF-*κ*B and MAPK pathways.

## 4. Discussion

With the changes to diet and lifestyle around the world, IBD has increasingly become a universal threat to human health [29]. Although the pathogenesis and development process of IBD are not completely clear, they are characterized as a barrier dysfunction caused by an excessive production of proinflammatory factors in mucosa and an increased permeability of the epithelial barrier [30]. Inflammatory cytokines, such as TNF, lead to the loss of barrier in the intestinal epithelial monolayer cultured in vitro, which is manifested in the activation and induction of cytoskeleton-mediated dysregulation of TJs, which in turn increase the production of TNF, resulting in the circulation of epithelial barrier dysfunction [4,31]. Therefore, inhibiting the release of inflammatory cytokines and regulating TJ function play an important role in restoring intestinal barrier function and maintaining disease remission [32]. At present, many bioactive polysaccharides have been shown to effectively alleviate mouse colitis and reduce the permeability of damaged Caco-2 cells [25,33,34,35]. Our previous study has shown that a water-soluble glucan (HSP-W) isolated and purified from HS showed immune-enhancing activity in vitro, but its functions of anti-inflammation and intestinal barrier protection have not been explored. Hence, we aimed to study the protective effect of HSP-W on the epithelial barrier in Caco-2 cells and its potential molecular mechanism. In the present study, we proved firstly that HSP-W could improve the intestinal epithelial barrier dysfunction induced by LPS through Caco-2 cell monolayer models, specifically reducing the LPS-induced enhancement in FD-4 flux and decrease in TEER. According to previous studies, LPS-induced intestinal epithelial barrier dysfunction is largely mediated by the induction of proinflammatory cytokines, including TNF-*α*, IL-6, and IL-1*β* [36,37]. Our results showed that HSP-W inhibited the inflammation of intestinal epithelial cells. TJs are the key determinants of mucosal barrier function, and a major determinant of cell bypass permeability in the absence of mucin deficiency or gross epithelial damage [32]. In our study, as expected, 50 μg/mL LPS significantly reduced the TJ protein expressions (Occludin, Claudin-1, and ZO-1), which was similar to the previous reports, indicating the change in cell permeability in inflammatory status [8,12,21]. However, based on our results, HSP-W significantly reversed this decrease, and further regulated intestinal epithelial barrier homeostasis and cell bypass permeability. Additionally, some previous studies found that the intestinal barrier function of mice with JAM-A deficiency decreased, and the proliferation and apoptosis rate of intestinal epithelial cells increased [38,39]. That is, the loss of the mucosal barrier will increase the severity of colitis [32]. In the present study, HSP-W also increased LPS-induced decreased expression of JAM-A protein. The above data indicate that HSP-W had potential activity to alleviate IBD.

Thereafter, the molecular mechanism of HSP-W protecting intestinal barrier function was explored using RNA-seq technology, a useful tool to study gene function and gene structure from the overall level and reveal specific biological processes and molecular mechanisms during the development of disease [40]. The results showed that, compared with the control group, LPS significantly increased the mRNA expression of MyD88 in Caco-2 cells, while HSP-W pretreatment could significantly reduce its expression. MyD88 is a significant adaptor protein of TLRs, the most intensively studied pattern recognition receptors (PRRS) in immunity, inflammation, and inflammatory diseases [41], and act as an important role in downstream signal transduction [42]. In this study, the KEGG pathway of DEGs between the LPS_SG and LPS groups was enriched in the “MAPK signaling pathway”. It is interesting that the enrichment analysis of those overlapped genes regulated reversely by HSP-W showed that the “Toll-like receptor signaling pathway” and the “NF-*κ*B signaling pathway” are also the important pathways for HSP-W to protect LPS-induced intestinal barrier injury.

Inflammatory responses are the result of the interaction between the immune system and damaged tissues, while the TLR4/MyD88/NF-*κ*B signaling pathway is significant for the regulation of inflammation and immunity [42,43]. TLR4 is one of the most intensively studied TLRs in human beings. It can initiate the dependent pathway by identifying endogenous molecules, such as LPS and DNA fragments, and by binding with MyD88 in cells [44]. NF-*κ*B is a key factor that triggers downstream inflammation, it is related to TJ protein expression as well as intestinal epithelial permeability and plays an important role in the development of IBD [45,46]. The downstream IKK activates phosphorylation of I*κ*B and promotes NF-*κ*B to translocate into the nucleus, thus promoting the release of proinflammatory cytokines (TNF-*α*, IL-6, and IL-1*β*) [47]. In turn, TNF-*α* activates NF-*κ*B, and initiates TNF-*α* transcription, to form a loop and aggravate inflammatory damage [48,49]. Therefore, proinflammatory-cytokines-induced activation of NF-*κ*B may be an important pathway of TJ barrier dysfunction. Our results showed that LPS could increase the protein expression of TLR4, p-I*κ*B, and NF-*κ*B p65, while HSP-W significantly inhibited LPS-induced phosphorylation of I*κ*B and activation of NF-*κ*B p65. According to the results of the transcriptome analysis, the effect of HSP-W on the nuclear translocation of NF-*κ*B was further tested through a nucleocytoplasmic separation test. The results showed that nuclear translocation of NF-*κ*B occurred after LPS stimulation, and the expression levels of downstream inflammatory cytokines, including TNF-*α*, IL-6, and IL-8, were also increased, whereas after HSP-W pretreatment, the content of NF-*κ*B and the levels of downstream inflammatory cytokines were significantly reduced. The above results further demonstrated that the protective effects of intestinal barrier function by HSP-W depends on the TLR4/MyD88/NF-*κ*B signaling pathway, through regulating the expression of TLR4, and inhibiting the phosphorylation of I*κ*B and activation of NF-*κ*B, thereby restraining the level of inflammatory cytokines and reducing the inflammatory response of Caco-2 cells stimulated by LPS.

**Figure 6:**
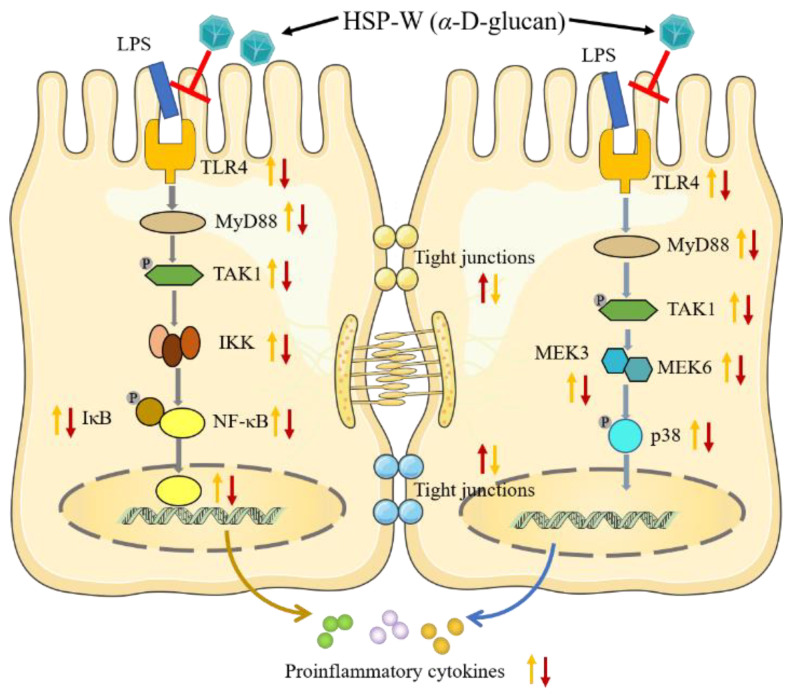
Schematic diagram summarizing the mechanisms underlying the protective effect of HSP-W against LPS-induced inflammation in Caco-2 cells.

Moreover, according to reported research, in addition to NF-*κ*B, excessive proinflammatory markers can also cause TJ barrier damage through the MAPK, MLCK, STAT, and AKT signaling pathways [15,50]. On the basis of our transcriptome results, the MAPK signal pathway is also the main pathway for HSP-W to repair LPS-induced intestinal barrier injury. The MAPK signaling pathway is mainly involved in cell growth, activation, division, environmental stress adaptation, inflammatory responses, and other important cell physiological processes [51]. At least four MAPKs have been found in mammalian intestinal cells, among which the ERK, JNK, and p38 MAPK pathways have been the most widely studied [52,53,54]. In previous studies, three MAPKs (p38 MAPK, JNK, and ERK1/ERK2) were affected in the intestinal mucosa of IBD patients. The activation of p38 MAPK and JNK protein was moderately increased, while the activation of ERK1/2 protein was significantly decreased [55]. What is noteworthy is that in the analysis of DEGs, we found that, among the genes related to MAPKs, only the p38-MAPK-pathway-related genes (MAPK14/13) were significantly down-regulated in the HSP-W treatment group. Therefore, in the subsequent experiments, Western blot was used to verify the p38 MAPK pathway. We could observe from the results that HSP-W could significantly inhibit p38 phosphorylation in inflammatory cells. Overall, HSP-W may inhibit the expression level of MyD88 through the down-regulation of TRL4, thereby inhibiting the activation of the p38 pathway to reduce the release of inflammatory factors and increase the expression level of TJ proteins in Caco-2 cells, leading to the protection of the intestinal barrier function, as illustrated in the mechanism diagram (Figure 6).

Therefore, based on the above results, it could be inferred that the molecular mechanism of HSP-W for intestinal barrier protection mainly involves the TLR4/MyD88/NF-*κ*B and p38 MAPK signaling pathways.

## 5. Conclusions

In conclusion, our results proved for the first time that polysaccharides from HS, such as HSP-W, may inhibit LPS-induced Caco-2 cell inflammation and up-regulate the expression of TJ protein through the MyD88-mediated NF-*κ*B and p38 MAPK signaling pathways, thereby reducing the permeability of the intestinal barrier and alleviating intestinal barrier damage. These findings further suggested the application of HS polysaccharide as a potential alternative or supplementary treatment for intestinal diseases.

## Data Availability

The data presented in this study are available on request from the corresponding author.

## Supplementary Materials

The following are available online at https://www.mdpi.com/article/10.3390/foods12030450/s1, Figure S1: The structure (possible repeated unit) of HSP-W, Table S1: Primer sequences of real-time PCR analysis.
