# Supplementary material for: Huangshui Polysaccharide Exerts Intestinal Barrier Protective Effects through the TLR4/MyD88/NF-κB and MAPK Signaling Pathways in Caco-2 Cells
Source: Foods. 2023 Jan 18;12(3):450. doi: 10.3390/foods12030450 (PMC9914309; doi:10.3390/foods12030450)
Supplement: Supplementary file 1 [file foods-12-00450-s001.zip › foods-2125406-supplementary.pdf]

Support Information

Huangshui Polysaccharide Exerts Intestinal Barrier Protective Effects through the TLR4/MyD88/NF-κB and MAPK Signaling Pathways in Caco-2 Cells

Jiaying Huo [1,2,3], Wenhao Pei [1,2], Guoying Liu [4], Weizheng Sun [3], Jihong Wu [1,2,*], Mingquan Huang [1,2], Wei Lu [4], Jinyuan Sun [1,2] and Baoguo Sun [1,2]

1 Key Laboratory of Brewing Molecular Engineering of China Light Industry, Beijing Technology and Business University, Beijing 100048, China
2 Beijing Laboratory of Food Quality and Safety, Beijing Technology and Business University, Beijing 100048, China
3 School of Food Science and Engineering, South China University of Technology, Guangzhou 510640, China
4 Anhui Gujing Distillery Co. Ltd., Bozhou 236820, China; liuguoying@gujing.com.cn (G.L.); luwei@gujing.com.cn (W.L.)

1. Chemical reagents

Lipopolysaccharide (LPS) was obtained from Sigma-Aldrich Co. (Beijing, China). Monosaccharide standards and glycine were from the same corporation with LPS. The culture medium and serum including DMEM and FBS were acquired from Gibco Life Technologies (Waltham, MA, USA). ELISA kits (IL-1β, IL-6, and TNF-α) were purchased from ABclonal Biotechnology Co., Ltd. (Wuhan, China). BCA protein kit and CCK-8 kit were provided by Shanghai yuanye Bio-Technology Co., Ltd (Shanghai, China). TBS380 Picogreen were obtainted from Invitrogen Life Technologies (California, USA). Hiseq2000 Truseq SBS Kit v3-HS, Miseq Reagent Kit V2 and Truseq™ RNA sample prep Kit were purchased from Illumina (San Diego, USA). Anti-Occludin (Ab216327), Anti-Claudin-1 (Ab211737), Anti-ZO-1 (Ab221546), Anti-JAM-A (Ab52647), and Anti-glyceraldehyde-3-phosphate dehydrogenase (GAPDH, Ab181602) were acquired from Abcam (Cambridge, UK).

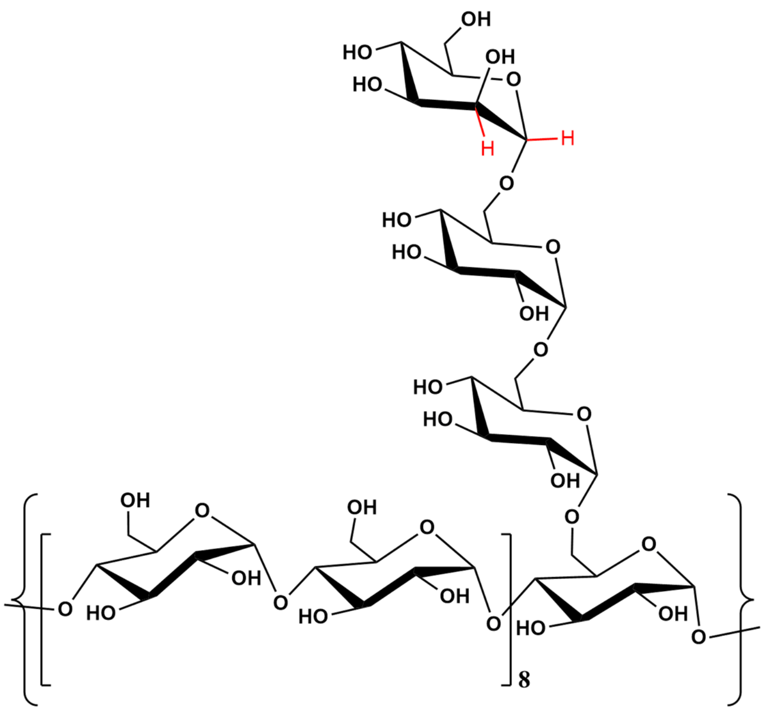

Figure S1 The structure (possible repeated unit) of HSP-W

Table S1 Primer sequences of real-time PCR analysis.

Primer	Name	Sequence (5'to3')
MyD88	MyD88-F	GCATATGCCTGAGCGTTTCG
	MyD88-R	TAGACCAGACACAGGTGCCA
IRAK4	IRAK4-F	GCCACCTGACTCCTCAAGTC
	IRAK4-R	TCCAAATCCTCCCTCTCCCA
LTB4R	LTB4R-F	CTCTGTCACTGCCCTGATGG
	LTB4R-R	GAGCGGTCTAGACTCATGGC
TIAF1	TIAF1-F	CCCTCTTTGTCTCAGCGTGT
	TIAF1-R	CCCAAGCCTTCCCTCTCAAG
GRHL2	GRHL2-F	ACCTGGAGAATTCCAAGCGG
	GRHL2-R	CACTCGTTGCTCTTCCCCAT
PRKCI	PRKCI-F	GGTCCGGGTGAAAGCCTAC
	PRKCI-R	TGTACACGGGTCTCCTTCCT
β-actin	β-actin-F	CATGTACGTTGCTATCCAGGC
	β-actin-R	CTCCTTAATGTCACGCACGAT
